# Risks and Benefits of Alcohol Use Over the Life Span

**Published:** 1996

**Authors:** Mary C. Dufour

**Affiliations:** Mary C. Dufour, M.D., M.P.H., is deputy director of the National Institute on Alcohol Abuse and Alcoholism, Bethesda, Maryland

**Keywords:** moderate AOD use, amount of AOD use, therapeutic drug effect, risk assessment, heart disorder, AODR (alcohol and other drug related) disorder, AODR mortality, age, traffic accident, impaired health, AOD associated consequences

## Abstract

Especially at low and moderate drinking levels, alcohol consumption can be associated with benefits (e.g., protection against coronary heart disease) as well as risks (e.g., increased risk of accidents). These benefits and risks may change across a person’s life span. To determine the likely net outcome of alcohol consumption, one must weigh the probable risks and benefits for each drinker. These assessments are based on the individual drinker’s consumption levels, his or her personal characteristics (e.g., age or preexisting risk factors for coronary heart disease), and subjective values as well as on social considerations. The validity of such assessments also depends on the accuracy with which alcohol consumption and alcohol-related consequences can be measured.

The consumption of alcoholic beverages has been a part of many cultures since at least the beginning of recorded history. Ancient texts from Persia, Egypt, Babylon, Greece, and China, as well as Biblical writers, have documented that people have been aware of alcohol’s beneficial and harmful effects for nearly as long as people have been drinking (Rubin and Thomas 1992). Alcohol consumption also is clearly a part of contemporary American life. As a result, although most people drink moderately and without ill effect, alcohol abuse and alcohol dependence are major health problems in the United States. In 1992 almost 14 million Americans age 18 and older met the criteria of the American Psychiatric Association’s *Diagnostic and Statistical Manual of Mental Disorders, Fourth Edition* (DSM–IV) for alcohol abuse and dependence (Grant et al. 1994). The alcohol-related costs to the Nation have been estimated to include 100,000 deaths and nearly $100 billion each year (Rice 1993).

Although moderate alcohol use has been sanctioned in the United States for a long time, its objective benefits have begun to be quantified only recently. For example, a substantial body of literature now exists describing the protective effects of low-level alcohol consumption against coronary heart disease, as evidenced primarily by the reduced risk of death from acute heart attacks (Klatsky et al. 1992).

The public also has become aware of alcohol’s general risks and benefits: News of grisly, alcohol-related, post-prom car crashes shares the media spotlight with reports on the cardioprotective effects of low-level alcohol consumption. Traffic statistics and news reports, however, do not answer the question, What are the health risks and benefits of alcohol consumption for a given person? This article defines alcohol-related risk, reviews several beneficial and detrimental consequences of alcohol consumption, and describes approaches to determining a person’s net risk or benefit from alcohol consumption.

## Defining Alcohol Consumption and Alcohol-Related Risk

Any discussion of the risks and benefits of alcohol consumption must address the issue of defining relevant terms. For example, few people would disagree that “excessive” drinking is harmful. Attempts to define “excessive” precisely, however, are likely to generate considerable discussion. Similarly, the terms “abstainer,” “light,” “moderate,” “heavy,” and “heavier” are commonly used to describe types of drinkers, although no standard definitions exist for these drinking levels. Many studies refer to respondents in the drinking category with the lowest mortality as “moderate drinkers” (Ferrence and Bondy 1994). Consequently, the definition of moderate drinking varies substantially across studies, ranging from less than one drink per day to three or more drinks per day (Ferrence and Bondy 1994). The definitions of what constitutes a “drink” are equally variable.

This definitional vagueness can lead to considerable confusion in interpreting epidemiological studies investigating the relationship between alcohol consumption and various risks and benefits. For example, people who intend to drink for cardioprotection need to know at what level of alcohol consumption such benefits accrue (i.e., how many and what size drinks constitute “moderate” drinking) and which additional risks are associated with that drinking level.

To be useful concepts, the risks and benefits associated with alcohol consumption also must be specified. For example, risks or benefits may be short term (i.e., acute), affecting the drinker within hours or days, or long term (i.e., chronic), exerting their effects over many years. Acute risks often arise from consumption of a large volume of alcohol in a short period of time. These risks include car crashes, violence, and alcohol poisoning as well as alcohol-medication interactions. Long-term risks include chronic diseases, such as alcohol dependence, alcoholic cirrhosis, and alcoholic heart muscle disease (i.e., cardiomyopathy). Among the acute benefits of alcohol consumption, improved mood (i.e., happiness and euphoria) probably is the most common (Dufour 1994). Alcohol’s cardioprotective effects appear to be both acute (e.g., decreased platelet adhesiveness in the blood, which reduces the danger of developing blood clots that may lead to heart attacks and strokes) and chronic (e.g., increased levels of high density lipoproteins, or “good” cholesterol) (Jackson et al. 1992). These examples illustrate the importance of specifying exactly which risks and benefits are associated with each alcohol-consumption level.

### The Concept of Net Outcome

The dichotomous view that alcohol is either only beneficial or only harmful is too simplistic; a more reasonable approach is the assessment of net outcome. This approach totals the positive and negative consequences of a person’s alcohol consumption to arrive at a net benefit or net risk for that person at his or her specific consumption level. For example, the determination of a net benefit implies that for a particular drinker, the benefits of drinking outweigh the risks.

A disadvantage of the concept of net outcome is that it assumes that one can separate the effects of alcohol from those of other confounding factors. Alcohol’s effects, however, cannot be considered in isolation. Some research suggests that moderate drinkers differ from abstainers and other drinkers not only in alcohol consumption but also in other health-related characteristics. For example, according to the Disease Prevention and Health Promotion Supplement to the 1985 U.S. National Health Interview Survey, moderate drinkers were more likely than either abstainers or other drinkers to sleep 7 to 8 hours each night, be at their ideal body weight, and exercise regularly (Dufour 1994). Each of these factors can contribute to good health, and their effects are difficult to disentangle from the effects of alcohol consumption per se.

The concept of net outcome requires a multidimensional frame of reference. For example, the relationships among the multiple factors that contribute to alcohol’s net effects (e.g., the drinker’s physical and psychological condition or social environment) may change over time. In addition, the assessment of correlations between current drinking levels and potential future consequences may be complicated by the fact that alcohol consumption is a dynamic process and can fluctuate over the short term (i.e., weeks) as well as over the long term (i.e., years). The implications of this variability can be illustrated best by using another medical parameter as an example: Blood pressure is extremely variable over the short term (i.e., the course of the day); nevertheless, a single blood pressure measurement fairly accurately reflects overall blood pressure at that point in time. Moreover, current hypertension could predict a worse outcome (e.g., an increased risk of a heart attack) in the future compared with normal blood pressure. Long-term changes in blood pressure (e.g., through treatment for hypertension), however, also would affect future outcome, making the risk and, therefore, the net outcome intermediate between normal blood pressure and untreated hypertension. By the same token, changes in alcohol consumption over the life span can influence certain associated risks and benefits and thus alter net outcome.

## Assessing Risks and Benefits of Alcohol Consumption

Because death is the easiest health indicator to assess, mortality—either from all causes combined or from specific causes—frequently is used to evaluate the health risks associated with certain drinking levels (see figure). (For information on assessing risk based on per capita alcohol consumption, see sidebar, pp. 148–149.) The findings of these analyses, however, are not always unequivocal. For example, Jackson and Beaglehole (1995) found that the risks associated with heavier drinking^1^ clearly outweighed the benefits associated with this level of alcohol consumption. In contrast, the risk-to-benefit balance for lower levels of alcohol consumption was not as obvious. On the one hand, light-to-moderate drinking appeared to reduce the relative risk of dying from coronary heart disease by as much as 50 percent, and light drinking also lowered the risk of death from ischemic stroke.^2^ On the other hand, light-to-moderate drinking increased mortality from cirrhosis, injury (e.g., suicides and car crashes), hemorrhagic stroke, breast cancer, and, possibly, bowel cancer.

**Assessing Alcohol-Associated Risks Based on Alcohol Consumption**

One indirect measure of alcohol-associated risks is per capita alcohol consumption. This approach is based on the observation that a person’s likelihood of experiencing negative consequences of drinking increases with the amount of alcohol consumed. Researchers are therefore closely monitoring trends in alcohol consumption.

***Recent Trends in Alcohol Consumption***

Following Prohibition, per capita alcohol consumption in the United States generally increased, reaching its peak in 1980 and 1981. Since then, alcohol consumption primarily has been declining. In 1993, the latest year for which complete data are available, per capita consumption of all alcoholic beverages combined reached its lowest level since 1964 (Williams et al. 1995). For the consumption of any kind of beverage, beer ranked fourth (behind soft drinks, coffee, and milk) in per capita consumption, a position it has held for many years. Beer also accounted for 57 percent of the absolute alcohol each person consumed; wine represented 13 percent; and spirits made up the remaining 30 percent, the lowest level for the consumption of spirits in 50 years (Williams et al. 1995).

To estimate total and per capita alcohol consumption, researchers rely on data such as alcoholic beverage sales, production, and tax revenues. Based on these data, the total apparent alcohol consumption for the United States in 1993 included approximately 5.8 billion gallons of beer, 454 million gallons of wine, and 341 million gallons of spirits (Williams et al. 1995). These amounts translate into 303 12-ounce cans of beer, 58 5-ounce glasses of wine, and 142 mixed drinks containing 1.5 ounces of distilled spirits for every man and woman age 14 and older in the country (Williams et al. 1995).

Although per capita alcohol consumption is a robust and useful measure of alcohol-consumption trends, it is only a relatively crude indicator of alcohol-related risk, because it assumes that every person in the population of interest drinks and that all people consume equal amounts of alcohol. In general, however, drinking patterns vary significantly among the members of a population.

***Drinking Patterns and Associated Outcomes***

Slightly more than one-half of American men and one-third of American women age 18 and older were current drinkers in 1992 (Dawson et al. 1995). Moreover, despite the fact that the legal drinking age is 21, alcohol use is common among young people. Approximately 56 percent of 8th graders, 71 percent of 10th graders, and 80 percent of 12th graders report having used alcohol at some time in their lives (Johnston et al. 1995). (For more information on the drinking patterns of adolescents and young adults, see the articles by Chassin and DeLucia, pp. 175–180, and Quigley and Marlatt, pp. 185–191.)

When evaluating individual risk based on these data, one must remember that actual alcohol consumption is unevenly distributed (see table) and that the 10 percent of drinkers who drink most heavily account for 50 percent of all alcohol consumed (Malin et al. 1982). The chances of experiencing negative consequences of drinking (e.g., accidents or medical problems) grow with increasing alcohol consumption. Accordingly, the remaining 90 percent of drinkers who drink only lightly or moderately should be at a small risk for a negative outcome (assuming, that is, that they do not consume their entire weekly alcohol allowance within a couple of hours on a Saturday night just before driving).

The assessment of individual risk is further complicated by the significant variability that exists in individual vulnerability to alcohol’s negative consequences. For example, not every heavy drinker will develop alcoholic cirrhosis. In fact, only 15 to 30 percent of the heaviest drinkers (i.e., alcoholics in treatment) are ever diagnosed with cirrhosis (Dufour et al. 1993). (For more information on potential problems associated with ascertaining the actual prevalence of cirrhosis, see the main article.) At the same time, a nonalcoholic woman consuming two drinks per day may develop cirrhosis (Dufour et al. 1993). These observations indicate that in addition to alcohol consumption, genetic and environmental factors play a critical role in determining individual outcome and risk.

—Mary C. Dufour

**Table:** Distribution of Drinking Levels Among Americans Age 18 and Older: National Longitudinal Alcohol Epidemiologic Survey, 1992

Drinking Status	Definition	Men (%)	Women (%)
Lifetime abstainer	Never had 12 drinks in any one year	22	45
Former drinker	Had 12+ drinks in 1 year but not in the year preceding the interview	22	21
Light drinker	Consumes < 0.22 oz of ethanol daily (> 1 drink per month but < 3 drinks per week)	19	17
Moderate drinker	Consumes 0.22–1.00 oz of ethanol daily (3–14 drinks per week)	23	13
Heavy drinker	Consumes > 1.00 oz of ethanol daily (> 14 drinks per week)	14	4

SOURCE: Adapted from Dawson et al. 1995.

ReferencesDawsonDAGrantBFChouPSGender differences in alcohol intakeHuntWAZakhariSStress, Gender, and Alcohol-Seeking BehaviorNational Institute on Alcohol Abuse and Alcoholism Research Monograph No. 29NIH Pub. No. 95–3893Bethesda, MDthe Institute1995321DufourMCStinsonFSCacesMFTrends in cirrhosis morbidity and mortality: United States, 1979–1988Seminars in Liver Disease1321091251993833760010.1055/s-2007-1007343JohnstonLDO’MalleyPMBachmanJGNational Survey Results on Drug Use From the Monitoring the Future Study, 1975–1994. Vol. 1: Secondary School StudentsNIH Pub. No. 95–4026Rockville, MDNational Institute on Drug Abuse19951151MalinHCoakleyJKaelberCMunchNHollandWAn epidemiologic perspective on alcohol use and abuse in the United StatesAlcohol Consumption and Related ProblemsNational Institute on Alcohol Abuse and Alcoholism Alcohol and Health Monograph No. 1DHHS Pub. No. (ADM)82–1190Washington, DCU.S. Govt. Print. Off198293153WilliamsGDStinsonFSStewartSLDufourMCSurveillance Report #35: Apparent Per Capita Alcohol Consumption: National, State, and Regional Trends, 1977–1993Bethesda, MDNational Institute on Alcohol Abuse and Alcoholism1995

The net outcome of all-cause mortality associated with a certain alcohol-consumption level therefore also depends on the drinker’s absolute risk of dying from these various causes. Accordingly, older people—who are at high absolute risk of coronary heart disease and ischemic stroke and at low risk for injury, cirrhosis, and other alcohol-related diseases—are most likely to benefit from low levels of alcohol consumption. In contrast, for men and women under age 40, who have relatively low absolute risk of dying from strokes, heart disease, and alcohol-related diseases but a high absolute risk of dying from injury, all-cause mortality will increase even at relatively low alcohol-consumption levels. For example, in a 15-year followup of 18- to 19-year-old Swedish male military conscripts, alcohol consumption lowered the risk of cardiovascular disease, but this disease only accounted for 4 percent of all deaths. Conversely, injury deaths, which accounted for 75 percent of all deaths, increased among drinkers, even among those drinking seven or fewer drinks per week (Andreasson et al. 1988). Finally, the absolute risk of death from injury or coronary heart disease is lower in young women than in young men, leading to an increase in all-cause mortality even in young women who are light drinkers (less than two drinks every 3 days) compared with abstainers (Fuchs et al. 1995). When interpreting these findings, however, one also must keep in mind that when researchers express alcohol consumption in terms of average drinks per day or per week, they often do not ascertain people’s actual drinking patterns. Thus, both a person having one drink each evening and a person having seven drinks on a Saturday night (i.e., a binge drinker) average seven drinks per week. Yet their risks of alcohol-related injuries will differ significantly, with a much higher risk for the binge drinker.

**Figure:**
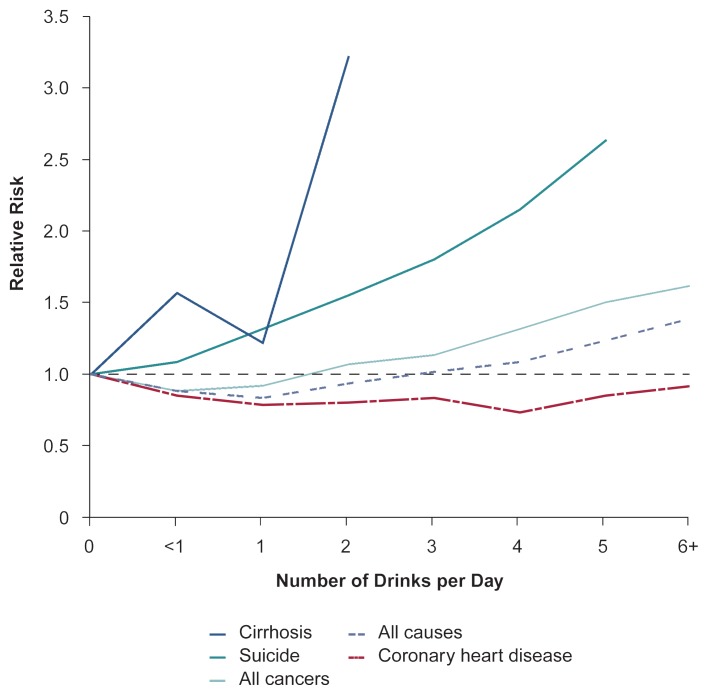
Relative risk of dying from various causes for middle-aged men consuming different alcohol amounts. More than 270,000 Caucasian men ages 40 to 59 were followed for 12 years; their death rates and causes of death were analyzed according to their alcohol-consumption levels. The relative risk is the ratio of the death rate from a specific cause among a certain group of drinkers to the death rate from the same cause among abstainers. A relative risk of less than 1.0 indicates a protective effect of alcohol consumption; a relative risk of greater than 1.0 indicates a detrimental alcohol effect. SOURCE: Bofetta and Garfinkel 1990.

These observations allow the following conclusions: First, for men in their forties and postmenopausal women, the mortality-related benefits of light-to-moderate drinking begin to outweigh the risks. Among women ages 50 to 70, however, all-cause mortality appears to be reduced only among those with at least one major risk factor for coronary heart disease (Fuchs et al. 1995). Second, the mortality-related benefits of low-level alcohol consumption continue to outweigh the risks for people in their sixties, seventies, and eighties (Jackson and Beaglehole 1995).

### Limitations to Risk and Benefit Assessment

Although it is clear that increasing levels of alcohol consumption are associated with greater physical and psychosocial hazards, the level at which the risks outweigh the benefits cannot be determined precisely. In assessing benefits and risks, the level of precision primarily depends on the supporting evidence that is available. To determine a specific risk or benefit associated with a given level of alcohol consumption, one must be able to measure accurately both the drinking level and the condition in question. Thus, significant underreporting of drinking levels could lead to an apparent increase in risk at low consumption levels.

Likewise, it may be difficult to ascertain the prevalence of particular alcohol-related conditions (e.g., cirrhosis). For example, autopsy studies suggest that as many as one-half of all cirrhosis patients remain asymptomatic throughout their lives. At present, a needle biopsy^3^ is the only definitive way to diagnose cirrhosis in a person without symptoms of liver disease. Thus, to ascertain the true incidence and prevalence of cirrhosis among drinkers with different consumption levels, one would need to perform liver biopsies on a large sample of “healthy” subjects. For ethical and technical reasons, such a study is not feasible. Accordingly, although it is clear that a correlation exists between alcohol consumption and cirrhosis, the exact amounts of alcohol—especially at lower consumption levels—that lead to cirrhosis cannot be determined accurately.

Other variables influencing the assessment of net outcome include the following:

- *Time:* What may be a health benefit at one point in time may be a health risk at another.
- *Subjective values:* What one person may perceive as a benefit, another person may perceive as neutral or harmful.
- *Social components:* The benefit to one person may be a risk to another person or to society as a whole.

### Examples of Risk-Benefit Assessments

A few examples can best illustrate how one can evaluate the specific benefits and risks associated with certain alcohol-consumption levels across different stages of the life span. Assessments such as these can help individuals and their health care providers estimate a person’s net outcome based on his or her combination of absolute risk factors. These estimates can then be used to develop individualized behavioral recommendations.

#### Risk Now Versus Benefit Later

For a 16-year-old boy, alcohol consumption may confer long-term cardioprotection; however, the boy’s chances of dying of a heart attack as a teenager are exceptionally small. On the other hand, alcohol-related traffic accidents are among the leading causes of death for teenagers (Dufour 1994). Drinking therefore produces a net risk for this boy.

#### Risk Now Versus Benefit and Risk Later

One of the hallmarks of alcohol dependence is loss of control over drinking; recovering alcoholics often cannot maintain moderate drinking patterns. Consequently, moderate drinking likely will escalate in this high-risk group to heavier drinking, with all its attendant risks for injury and chronic disease. Therefore, moderate alcohol consumption poses a net health risk for recovering alcoholics, even though it also may have cardioprotective effects.

#### Benefit Now and Later

A 55-year-old postmenopausal woman who has risk factors for heart disease may benefit from alcohol’s cardioprotective effects. If she takes no contraindicated medications, low-level alcohol consumption may confer a net benefit to her.

#### Benefit Now Versus Risk Now

For a 55-year-old postmenopausal women with risk factors for heart disease and a strong family history of breast cancer, assessment is more difficult. Heart disease is the leading cause of death for American women in that age group, and breast cancer is the second most common cause of cancer deaths among women (National Center for Health Statistics 1993). Currently, it is unknown whether moderate drinking would confer a net risk or a net benefit for the woman in question, because the same low consumption levels have been associated with both alcohol’s cardioprotective effects and increased risk of breast cancer. Once researchers have elucidated the exact mechanisms by which alcohol contributes to cardioprotection and breast cancer, a more accurate assessment of this woman’s net outcome will be possible.

#### Benefit to One Person Versus Harm to Another

For a woman early in the first trimester of pregnancy, a few drinks most likely will have no net harmful effects for the woman herself. This level of alcohol consumption, however, may have serious negative consequences for the developing fetus. Thus, maternal drinking during pregnancy constitutes a net risk for the fetus. Currently, one cannot predict whether a given fetus will be injured by a specific alcohol amount or whether a safe threshold of alcohol consumption during pregnancy exists below which no fetal damage occurs. Until researchers can answer these two questions, the safest course is for women to avoid all alcohol consumption during pregnancy.

## Conclusions

The ultimate goal of assessing the risks and benefits associated with alcohol consumption is to provide recommendations that promote a healthy lifestyle and thus extend people’s lives. Yet despite all the advances in medical and genetic research, the effects of these recommendations on the individual cannot be determined. Another medical example illustrates this point. Tsevat and colleagues (1991) have calculated that if people changed their behavior to eliminate *all* heart disease, the average life expectancy would increase by 3.1 years for a 35-year-old man and by 3.3 years for a 35-year-old woman. However, these 3 added years of life are just a statistical average. The actual benefits of a healthy lifestyle to a given person may be far greater or far less and cannot be predicted in advance.

With these qualifications in mind, assessment of alcohol-associated health risks and benefits leads to the following alcohol-consumption recommendations:

- For certain groups of people, alcohol consumption is associated with a net health risk, and thus abstinence is the safest course. These groups include women who are pregnant or trying to conceive, recovering alcoholics, people about to operate a motor vehicle or other dangerous machinery, and people having medical contraindications or taking medications that interact with alcohol.
- Middle-aged and older adults who do not fall into any of the abovementioned exclusionary categories and who enjoy consuming alcohol in moderation are likely to experience net health benefits.
- Heavier drinkers likely will benefit from moderating their consumption.

The *Dietary Guidelines for Americans* (U.S. Department of Agriculture and Department of Health and Human Services 1995) recommends drinking levels of no more than one drink^4^ per day for women and two drinks per day for men. Because each person’s circumstances vary, however, people should discuss these recommendations with their physicians or other health care providers.

Finally, although the prospect of net health benefits from moderate drinking may appeal to people, it generally is not the only or primary reason for drinking. Instead, most people drink because they like alcohol’s taste, effects, or both. The remaining articles in this journal issue discuss in more detail the reasons for and consequences of drinking across the life span.

## Footnotes

1 In their study, Jackson and Beaglehole (1995) defined “heavier” drinking as four or more drinks per day. Accordingly, “light-to moderate” drinking was defined as up to three drinks per day.

2 An ischemic stroke is caused by a blocked blood vessel in the brain. A hemorrhagic stroke is caused by bleeding from a blood vessel in the brain.

3 A needle biopsy is a procedure in which a hollow needle is inserted through the patient’s skin into the organ under investigation (e.g., the liver) to retrieve a small tissue sample for further examination.

4 A drink is defined here as 12 ounces of beer, 5 ounces of wine, or 1.5 ounces of distilled spirits.
